# Dynamic brain lateralization patterns in Chinese naturalistic language comprehension and association with sex differences: a 7T functional magnetic resonance imaging study

**DOI:** 10.1093/psyrad/kkag003

**Published:** 2026-01-20

**Authors:** Ruohan Zhang, Shujie Geng, Xiaoqing Zheng, Wanwan Guo, Chun-Yi Zac Lo, Jiaying Zhang, Xiao Chang, Xinran Wu, Yufeng Zhang, Jie Zhang, Miao Cao, Jianfeng Feng

**Affiliations:** Warwick Manufacturing Group, University of Warwick, Coventry CV4 7AL, UK; Institute of Science and Technology for Brain-Inspired Intelligence, Fudan University, Shanghai 200433, China; Key Laboratory of Computational Neuroscience and Brain-Inspired Intelligence (Fudan University), Ministry of Education, China; School of Computer Science, Fudan University, Shanghai 200433, China; Institute of Science and Technology for Brain-Inspired Intelligence, Fudan University, Shanghai 200433, China; Key Laboratory of Computational Neuroscience and Brain-Inspired Intelligence (Fudan University), Ministry of Education, China; Institute of Intelligent Bioelectrical Engineering, National Yang Ming Chiao Tung University, Hsinchu 300093, Taiwan; School of Artificial Intelligence, Beijing University of Posts and Telecommunications, Beijing 100876, China; State Key Laboratory of Cognitive Neuroscience and Learning, Beijing Key Laboratory of Brain Imaging and Connectomics, IDG/McGovern Institute for Brain Research, Beijing Normal University, Beijing 100875, China; Institute of Science and Technology for Brain-Inspired Intelligence, Fudan University, Shanghai 200433, China; Key Laboratory of Computational Neuroscience and Brain-Inspired Intelligence (Fudan University), Ministry of Education, China; Institute of Science and Technology for Brain-Inspired Intelligence, Fudan University, Shanghai 200433, China; Key Laboratory of Computational Neuroscience and Brain-Inspired Intelligence (Fudan University), Ministry of Education, China; Department of Chinese Language and literature, Fudan University, Shanghai 200433, China; Institute of Science and Technology for Brain-Inspired Intelligence, Fudan University, Shanghai 200433, China; Key Laboratory of Computational Neuroscience and Brain-Inspired Intelligence (Fudan University), Ministry of Education, China; Institute of Science and Technology for Brain-Inspired Intelligence, Fudan University, Shanghai 200433, China; Key Laboratory of Computational Neuroscience and Brain-Inspired Intelligence (Fudan University), Ministry of Education, China; Institute of Science and Technology for Brain-Inspired Intelligence, Fudan University, Shanghai 200433, China; Key Laboratory of Computational Neuroscience and Brain-Inspired Intelligence (Fudan University), Ministry of Education, China; Department of Computer Science, University of Warwick, Coventry CV4 7AL, UK

**Keywords:** dynamic laterality, Chinese language processing, naturalistic imaging, functional states, sex differences, sex hormones

## Abstract

**Background:**

Although language is traditionally regarded as unique to humans and predominantly left-lateralized in the brain, the dynamic interplay between cerebral hemispheres during language processing remains poorly understood.

**Methods:**

Using 400 functional magnetic resonance imaging scans acquired with a 7T scanner under diverse narrative stimuli, this study examined whole-brain functional dynamic lateralization patterns during Chinese language processing and explored potential sex differences.

**Results:**

We identified two distinct dynamic lateralization states. While core language regions consistently showed left-lateralization, other brain regions displayed reversed lateralization. These two states—characterized by higher-level functional regions lateralizing either left or right—corresponded to the processing of rational and emotional content, respectively. Notably, males showed a stronger tendency toward the former state, whereas females inclined toward the latter, particularly during the processing of rational content. Genetic analyses further suggested that sex differences in these lateralization states may be influenced by sex hormones.

**Conclusion:**

This study offers novel insights into the dynamic organization of cerebral lateralization during Chinese language processing.

## Introduction

Languages are distinctively used by humans, and language processing is characterized by intricate, complex, and diverse neural mechanisms. One notable aspect of language processing is brain lateralization, as the language system was traditionally assumed to be situated mainly in the left hemisphere, enhancing efficiency in multitask performance (Vallortigara *et al*., 2011; Esteves *et al*., 2020) and reducing redundancy in neural organization (Lazard *et al*., 2012; McAvoy *et al*., 2015; Joliot *et al*., 2016; Gerrits *et al*., 2020). Nevertheless, some neuroimaging studies have revealed language processing-related regions in the right hemisphere, though they are relatively marginal (Binder *et al*., 1997; Fedorenko *et al*., 2010; Price, 2012). Furthermore, as a higher-level cognitive function, language processing relies on information coordination across a broad range of cognitive domains, including memory, learning, and cognitive control (Bialystok *et al*., 2004; Novick *et al*., 2010; Doron *et al*., 2012; Makuuchi and Friederici, 2013; Fedorenko and Thompson-Schill, 2014; Fen *et al*., 2015; Federmeier *et al*., 2020; Pliatsikas, 2020; Zheng *et al*., 2020; Peng *et al*., 2023). Most previous studies on language lateralization typically considered language processing to be a distinct module within the brain. However, the functional lateralization of language processing throughout the brain cortex remains largely unknown.

Moreover, lateralization has traditionally been treated as a static feature of the human brain. However, researchers have recognized that the inherent dynamic coordination of information is a fundamental requirement for optimal brain functioning, spanning both cognitive and behavioural domains (Hiltunen *et al*., 2014; Kucyi *et al*., 2018; Wirsich *et al*., 2020). Specifically, functional magnetic resonance imaging (fMRI) studies have revealed that language processing involves highly dynamic interactions across different brain regions (Doron *et al*., 2012; Fedorenko and Thompson-Schill, 2014; Feng *et al*., 2015; Pliatsikas, 2020; Zheng *et al*., 2020; Peng *et al*., 2023). The processing hierarchy from visual processing to working memory and core language systems during sentence recognition has been revealed with dynamic causal models (Makuuchi and Friederici, 2013). Different dynamic interaction patterns between linguistic and domain-general systems during various cross-language processing operations, such as backwards and forwards translation, have also been reported (Zheng *et al*., 2020). Notably, by examining the dynamic properties of different brain regions within a predefined language system, researchers found that regions in the left hemisphere have stable processing functions, while those in the right hemisphere have more flexible periphery functions (Chai *et al*., 2016). Nevertheless, our understanding of the dynamic functional lateralization of language processing across the entire cerebral cortex remains limited. In a recent study, Wu *et al*. (2022) investigated dynamic lateralization in the brain in the resting state and its association with language functions and cognitive flexibility. This work suggests the potential of exploring dynamic lateralization across the whole brain during language processing.

Furthermore, sex differences have been reported for many cognitive functions, such as theory of mind (Greenberg *et al*., 2023), visual–spatial processing (Clements *et al*., 2006), and language processing (Shaywitz *et al*., 1995; Kansaku *et al*., 2000; Clements *et al*., 2006; Xu *et al*., 2020; Yang *et al*., 2020). Brain imaging studies have suggested sex differences in brain lateralization during language processing. For instance, a few investigations have indicated that males exhibit stronger left lateralization in the brain than females while performing language tasks (Levy, 1969; Kimura, 1992; Shaywitz *et al*., 1995), while females exhibit greater bilateral activation in language-related regions than males, including the inferior frontal gyrus, posterior superior temporal gyrus, and fusiform gyrus (Kansaku *et al*., 2000; Clements *et al*., 2006; Burman *et al*., 2013; Xu *et al*., 2020; Yang *et al*., 2020). Nevertheless, the impact of sex on dynamic lateralization patterns throughout the brain during language processing remains largely unknown. Additionally, although studies on semantics have revealed distinct patterns of brain activation across cortical brain regions in response to various language contents (Huth *et al*., 2016; Bi, 2021), the potential impact on dynamic lateralization, especially in the different sexes, remains unexplored.

**Figure 1:**
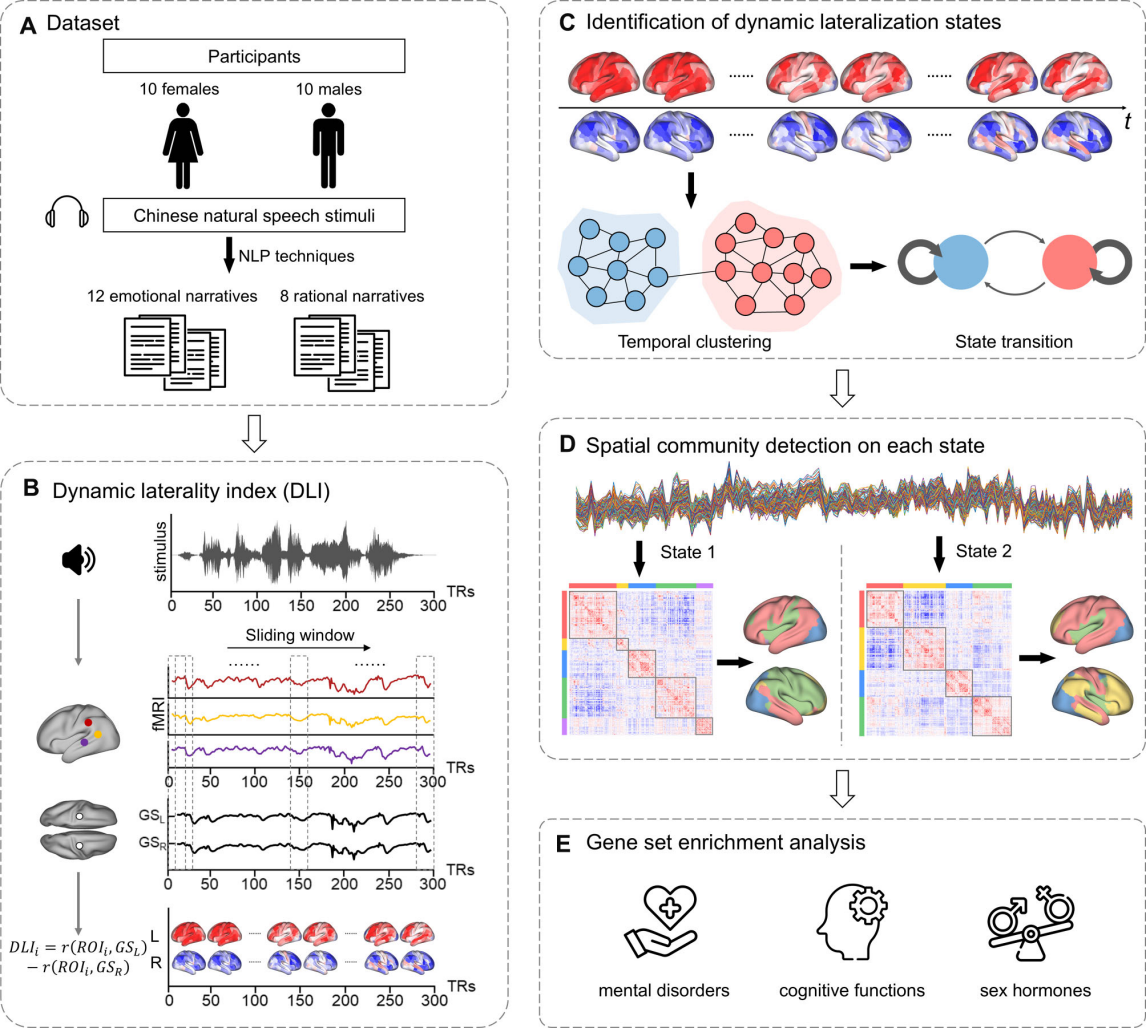
Overview of the study. (**A**) Task-based fMRI data acquired from a total of 20 participants, including 10 females. Participants were presented with a series of 20 8- to 12-min narratives, which were categorized into two groups (12 emotional narratives and eight rational narratives) using natural language processing (NLP) techniques and a hierarchical clustering algorithm. (**B**) The definition of the dynamic laterality index (DLI). The DLI of the region of interest (*ROI_i_*) within the time window *t* is defined as the correlation coefficient (*z*-transformed) between global signal of the left hemisphere (*GS_L_*) and the ROI minus the correlation coefficient between global signal of the left hemisphere (*GS_R_*) and the ROI. Using a sliding window approach, we obtained a DLI time series for each ROI. (**C**) The temporal clustering of the whole-brain lateralization patterns, which identifies recurring dynamic lateralization states. (**D**) The spatial clustering results of the dynamic lateralization states, which identified spatial communities. (**E**) The gene set enrichment analysis of the dynamic lateralization states.

To address these issues, we acquired fMRI data using an ultrahigh-field 7T Siemens Terra MR scanner with 400 fMRI scans of diverse Chinese narrative stimuli from 20 participants (with a mean age of 23.7 ± 1.8 years and 10 females for sex balance). During the task, all participants listened to narratives that replicated authentic language to simulate language processing in real-life situations. For the analyses, we employed a data-driven approach that combines a sliding window technique with the global signal-based laterality index to identify recurring whole-brain dynamic lateralization states. Then, we examined the spatial and temporal properties of the states as well as influences of sex and content. Next, we explored the genetic underpinnings of the observed dynamic lateralization states with publicly available postmortem brain-wide gene expression data. Finally, using resting-state fMRI data from the Human Connectome Project (HCP) cohort, we investigated whether the properties of the dynamic lateralization states observed during the Chinese natural listening task are related to inherent lateralization patterns. Figure 1 provides a general schema of the present study.

## Materials and Methods

### Participants and task-based fMRI dataset

Fully informed consent was obtained from all participants by Fudan University, and the research procedures and ethical guidelines were approved by the Fudan University institutional review board. The ‘Narratives’ collection consists of task-based fMRI datasets acquired while 20 participants listened to naturalistic spoken stories, totalling 400 functional scans. All participants were healthy and had normal hearing (mean age 23.7 ± 1.8 years, 10 females, all right-handed). The participants were postgraduate students with comparable educational levels and similar language proficiency and comprehension abilities, ensuring a relatively homogeneous educational background. The task-based fMRI data were acquired using an ultrahigh-field 7T Siemens Terra MR scanner at the Zhangjiang International Brain Imaging Centre (ZIC) of Fudan University, Shanghai, China. A 32-channel Siemens volume coil (Siemens Healthcare) was used for data acquisition. Functional scans were obtained using an echo planar imaging (EPI) sequence with the following parameters: repetition time (TR) = 1 500 ms, echo time (TE) = 25 ms, flip angle = 65°, voxel size = 1.5 × 1.5 × 1.5 mm, matrix size = 128 × 128, field of view (FOV) = 1920 × 1920 mm, slice thickness = 1.5 mm, and number of slices = 96. The T1-weighted anatomical data were collected on a 3T scanner with the following parameters: TR = 2500 ms, TE = 2.22 ms, flip angle = 8°, matrix size = 300 × 320, FOV = 240 × 256 mm, slice thickness = 0.8 mm, and number of slices = 208.

### Data preprocessing

Image preprocessing was performed using Statistical Parametric Mapping-12 (SPM12; http://www.fil.ion.ucl.ac.uk/spm). To ensure T1 equilibrium, several volumes were not recorded prior to the trigger. The volumes were temporally realigned to the middle EPI volume and spatially realigned to correct for head movement. The structural images of each participant were registered to the mean EPI image, segmented, and normalized to Montreal Neurologic Institute (MNI) space. The realigned EPI volumes were then normalized to MNI space using deformation field parameters obtained from the structural image normalization. Finally, the normalized EPI volumes were spatially smoothed using a 6 × 6 × 6-mm Gaussian kernel and high-pass filtered to reduce noise. After preprocessing, the cortical gray matter was parcellated into 426 regions, including 360 cortical areas and 66 subcortical areas, using the HCPex atlas (Huang *et al*., 2022). The HCPex atlas is a modified and extended version of the surface-based Human Connectome Project-MultiModal Parcellation atlas of human cortical areas (HCP-MMP v1.0) (Glasser *et al*., 2016). This atlas provides a detailed and multimodal parcellation of brain regions, enabling the examination of dynamic lateralization in different brain regions. For each participant, time series were extracted by calculating the mean signal of all voxels within each of the 426 brain regions.

### Chinese natural speech stimuli

In the task, a set of 20 8- to 12-min narratives were utilized as the Chinese natural speech stimuli. These narratives covered a wide range of genres, including fairy tales, fables, novels, essays, news, and scientific essays. All narratives were drawn from commonly available Chinese literature, news, and essays but were confirmed as unfamiliar to participants prior to scanning. Comprehension was checked following scans via brief summaries. Texts were balanced in length, lexical complexity, and topic diversity to minimize difficulty-related confounds. A detailed summary of each narrative is presented in Table S1. Each participant completed 20 separate narrative-listening sessions (one per narrative). Each session constituted a full fMRI run conducted on different days to avoid fatigue. The duration of each scan was customized to match the length of the corresponding narrative and included a period of silence lasting 2 s both before and after the narrative was presented. For each participant, the fMRI scans were conducted on different days. The presentation order of the 20 narratives was pseudo-randomized across participants to avoid systematic order effects and balance emotional/rational content exposure across sessions, with a total of ~2.7 h of scanning time. The narratives were played to participants using Sensimetrics S14 in-ear piezoelectric headphones.

### Classification of Chinese natural speech stimuli

Data-driven techniques incorporating natural language processing techniques and the hierarchical clustering algorithm were employed to classify the Chinese natural speech stimuli. The natural language processing (NLP) techniques applied in this analysis included the ‘Jieba’ Chinese text segmentation system and text vectorization. First, Chinese word segmentation was performed using the *Jieba* word segmentation tool (https://github.com/fxsjy/jieba/). This tool divided continuous sentences into words. Next, each word was represented as a 300-dimensional numerical vector based on pretrained Chinese word vectors (Li *et al*., 2018). These vectors were trained with rich content features (word, ngram, and character) and large corpora, including Baidu Encyclopedia, Wikipedia_zh, Wikipedia_zh, Sogou News, Financial News, Zhihu_QA, Weibo, and Literature. This training produced accurate word representations in a high-dimensional semantic space. Subsequently, each narrative was represented as the term frequency–inverse document frequency (TF-IDF) weighted sum of the embedding vectors corresponding to each word. The TF-IDF is a statistical method that calculates the significance of a token or word to a document within a set of documents (Hakim *et al*., 2014). The TF-IDF score combines the term frequency (TF) and inverse document frequency (IDF). The TF, tf(*t, d*), measures how many times a token or word appears in a document:


\begin{eqnarray*}
{\mathrm{tf}}\left( {t,d} \right) = \frac{{{{f}_{t,d}}}}{{\mathop \sum \nolimits_{t^{\prime}\in d} {{f}_{t^{\prime},d}}}},
\end{eqnarray*}

where *f_t,d_* is the number of times word *t* occurs in document *d*, and the sum of *f*_*t*′,*d*_ represents the total number of words in document *d*.

The IDF measures how frequent or rare a token is within the entire document set:


\begin{eqnarray*}
{\mathrm{idf}}\left( {t,D} \right) = {\mathrm{log}}\frac{N}{{\left| {\left\{ {d\in D:t\in d} \right\}} \right|}}
\end{eqnarray*}

where *N* is the total number of documents and $| {\{ {d\in D:t\in d} \}} |$ denotes the number of documents in which the word appears.

By multiplying these two metrics, a TF-IDF score is obtained for each word in a document:


\begin{eqnarray*}
{\mathrm{tf - idf}}\left( {t,d,D} \right) = {\mathrm{tf}}\left( {t,d} \right) \cdot {\mathrm{idf}}\left( {t,D} \right)
\end{eqnarray*}

Finally, the hierarchical clustering algorithm (Nielsen, 2016) was applied to the set of 20 vectorized narratives. In this algorithm, the Euclidean distance and inverse squared distance (i.e. minimum variance algorithm) are utilized to calculate the distances between clusters. The clustering results were visualized using a dendrogram. The optimal number of clusters was determined by considering both the structure of the dendrogram and the silhouette criterion (Rousseeuw, 1987). The clustering labels obtained with this classification algorithm were then used to investigate differences in dynamic lateralization when processing different types of Chinese natural speech stimuli.

### Dynamic laterality index

To assess the dynamic brain lateralization during Chinese natural speech processing, we adopted a dynamic laterality index (DLI) framework combined with a sliding-window approach, as proposed by Wu *et al*. (2022). The DLI quantifies the temporal fluctuation of hemispheric dominance in functional integration. Unlike traditional lateralization indices based on activation contrasts between homologous voxels, DLI reflects how strongly each region dynamically couples with the left or right hemispheric global signal (GS) over time, providing a time-resolved measure of hemispheric coordination. Following this framework, we computed the DLI within successive overlapping windows of the fMRI time series. Specifically, using a sliding window of 20 TRs in length and a step size of 1 TR, we calculated for each region *i*:


\begin{eqnarray*}
DL{{I}_i} = r\left( {RO{{I}_i},G{{S}_L}} \right) - r\left( {RO{{I}_i},G{{S}_R}} \right),
\end{eqnarray*}

where *ROI_i_* denotes the blood oxygen level-dependent (BOLD) time series of regions of interet (*ROI_i_*), and *GS_L_* and *GS_R_* denote the mean global signal, i.e. the BOLD time series averaged across all voxels, of all regions within the left and the right hemispheres. The Pearson correlation coefficient *r* is Fisher-z-transformed. To test the reproducibility of the DLI, we also employed two other window lengths to evaluate the impact of the window length on the DLI results.

To quantify the magnitude of the time-varying laterality, we measured the laterality fluctuation (LF), which is defined as the standard deviation of the laterality time series (Wu *et al*., 2022), of each ROI for every participant.

### Identification of dynamic lateralization states

To identify recurring whole-brain dynamic lateralization states, a two-stage temporal clustering approach was employed. In the first stage, we focused on individual-level analysis. For each participant, a temporal similarity matrix was computed across the total 6114 time windows of the 20 scanning sessions. The temporal similarity was measured as the Pearson correlation between each pair of whole-brain dynamic lateralization states in the different time windows. Then, we applied the Louvain community detection algorithm (Blondel *et al*., 2008) to the temporal similarity matrix of the 6114 dynamic lateralization time windows for each participant to generate clusters. The γ parameter was set to 1 in the community detection process, and the algorithm was stopped when the modularity Q no longer improved (i.e. improvement <1.0 × 10^−100^), ensuring the best cluster partitioning. Each cluster represented a dynamic lateralization state at the individual level, and the state centroids (i.e. the mean of whole-brain dynamic lateralization state within a cluster) were calculated.

In the second stage, we focused on group-level analysis. To obtain group-level recurring dynamic lateralization states, a temporal similarity matrix was computed using the state centroids from all participants. The Louvain community detection algorithm was applied to analyse this matrix. Based on the group-level clustering results, all the dynamic lateralization time windows of all participants were reclassified accordingly.

### Mapping text sentiment to dynamic lateralization states

#### Audio text segmentation and sentiment labelling

The audio data were segmented into sentences using NLP techniques. Regular expression operations were employed to identify sentence boundaries in the audio transcriptions, followed by manual correction. A group of four participants (two females and two males) was recruited to assign sentiment labels to the sentences. These participants were evenly distributed between sexes and were not involved in the task-based fMRI scanning. They rated the sentiment of each sentence, distinguishing between emotional (marked as 1) and rational (marked as 0) sentences. The sentiment ratings provided by the four participants were then averaged to obtain a sentiment score for each sentence. Four raters (two males and two females) independently performed the sentiment labelling. Inter-rater reliability indicated substantial agreement among raters (Cohen’s κ = 0.75) (Landis and Koch, 1977). Moreover, we determined the sentiment score of each individual Chinese character within the sentences according to the score of that sentence.

#### Mapping audio texts to functional brain imaging windows

The time windows defined in the task-based fMRI data and the corresponding sections of the audio data were aligned by synchronizing the timestamps of the fMRI data with the timestamps associated with each sentence in the audio data. This temporal alignment enabled precise correspondence between the linguistic information and the neural activity captured in each window.

#### Calculation of sentiment scores for dynamic lateralization states

We collected the Chinese characters in the audio data for each time window in the task-based fMRI data. Subsequently, the sentiment score for each time window was computed by averaging the scores of all Chinese characters within the window, which served as a measure of the emotion of the contents conveyed by the audio data during that particular window. Moreover, to minimize the influence of individual differences on the lateralization state, we determined the lateralization state of each time window by measuring the mode across all participants. This approach established a relationship between the content emotions (emotional or rational) in the audio texts and the corresponding dynamic lateralization states.

### Spatial properties of dynamic lateralization states

#### Community structure of each state

To investigate the spatial organization of the dynamic lateralization states, we first extracted the lateralization time series for each state. The lateralization time series for each state were obtained by concatenating the data from all scanning sessions of all participants. Subsequently, we calculated a spatial similarity matrix (426 × 426) based on the lateralization time series across the entire brain for each state. The similarity between regions was estimated using the Pearson correlation coefficient, and only relationships with positive weights were retained. Next, we employed the Louvain community detection algorithm (Blondel *et al*., 2008) to determine the community structure of each dynamic lateralization state based on the spatial similarity matrix. During the community detection process, we set the parameter γ to 1, and the algorithm iterations were stopped when the modularity Q no longer improved (i.e. improvement <1.0 × 10^−100^) to obtain the best community partitioning results. To ensure comparability among communities across different dynamic lateralization states, we aligned the communities by relabelling them according to their anatomical locations.

Additionally, we calculated the mean DLI and mean activation values of the communities for each state. Because the language listening task was a continuous process without contrasting tasks, we set the activation threshold as the mean BOLD signal value across the whole brain at each time point. The activation value for each brain region was obtained by thresholding the BOLD signal values.

#### Community segmentation and integration during state transitions

To quantify the process of community change during state transitions, we introduced a community transition rate (CTR) matrix that captures the segmentation and integration of communities during state transitions. Assuming a state space *S*, the CTR matrix from state *i* with *M* communities to state *j* with *N* communities is defined as:


\begin{eqnarray*}
CT{{R}^{ij}} = \left(
\begin{array}{c{@}{\quad}c{@}{\quad}c{@}{\quad}c}
{T_{11}^{ij}}&{T_{12}^{ij}}&\cdots &{T_{1N}^{ij}}\\
{T_{21}^{ij}}&{T_{22}^{ij}}&\cdots &{T_{2N}^{ij}} \\
\vdots & \vdots & \ddots &\vdots \\
{T_{M1}^{ij}}&{T_{M2}^{ij}}&\vdots &{T_{MN}^{ij}}
\end{array}
\right),i,{\mathrm{\ }}j \in S.
\end{eqnarray*}

Here, $T_{mn}^{ij}$ represents the (*m, n*) element of *CTR^ij^*, given by:


\begin{eqnarray*}
CT{{R}^{ij}}\left( {m,n} \right) = T_{mn}^{ij} = \frac{{N{{R}_{mi}}}}{{N{{R}_{nj}}}},
\end{eqnarray*}

$m = 1,{\mathrm{\ }}2,{\mathrm{\ }} \cdots ,M{\mathrm{\ }}and{\mathrm{\ }}n = 1,{\mathrm{\ }}2, \cdots ,{\mathrm{\ }}N.$

where *NR_mi_* represents the number of brain regions in the *m*th community in state *i*, and *NR_nj_* represents the number of brain regions in the *n*th community in state *j*. Since the matrix elements represent transition rates, they must satisfy:


\begin{eqnarray*}
0 \leq CTR_{ij} (m,n) \leq 1.
\end{eqnarray*}

Furthermore, as the brain regions in a community in one state must be allocated to one of the available communities in another state, the transition matrix must satisfy:


\begin{eqnarray*}
\mathop \sum \limits_{n\ = \ 1}^N CT{{R}_{ij}}( {m,n} ) = 1,\,\, {{\rm for\,\, all}\,\, m.}
\end{eqnarray*}

### Temporal properties of dynamic lateralization states

#### Occurrence rate and mean dwell time

The temporal properties of the dynamic lateralization states were assessed using the occurrence rate and mean dwell time (Ryali *et al*., 2016; Zhou *et al*., 2019). The occurrence rate represents the proportion of time windows spent in each dynamic lateralization state, measured as a percentage. The mean dwell time indicates the duration of time that a participant remains in a specific dynamic lateralization state, calculated by averaging the number of consecutive windows spent in that state before transitioning to another state.

#### Transition probability

To characterize the transitions between brain states, we estimated the transition probability matrix using the dynamic lateralization state time series for each participant and scanning session. Assuming a state space *S* with *N* brain states, the estimated transition probability matrix for the dynamic lateralization state series is denoted as *PN* × *N*. The element (*i*,  *j*) in matrix *P* is calculated as:


\begin{eqnarray*}
{{p}_{ij}} = \frac{{{{n}_{ij}}}}{{\mathop \sum \nolimits_j {{n}_{ij}}}},i,j\in S
\end{eqnarray*}

where *n_ij_* denotes the number of times state *i* transitions to state *j*.

### Statistical analysis

We employed linear mixed-effects models (Pinheiro and Bates, 1996) to investigate the potential effects of the temporal properties of the dynamic lateralization states, with age and the number of time windows as covariates in the analysis.

#### Differences in the temporal properties between states

We compared the differences in the occurrence rate among different states using the following model:


\begin{eqnarray*}
occurrence\ \textit{rate}&\sim & \textit{state} + ( {1{\mathrm{|}}\textit{subject}\ ID} ) + ( {1{\mathrm{|}}sex} ) \\ &&+\, ( 1{\mathrm{|}}\textit{content}\ \textit{types} ).
\end{eqnarray*}

This model included fixed effects for brain lateralization states and random effects for participants, sex, and content types since the task employed a within-subject design. To assess the significance of the explanatory variable in the mixed-effects model, we performed *N* = 10 000 permutations of the response variable. Additionally, we evaluated the differences in the mean dwell time among different states using the same model.

#### Differences in the transition probabilities between transition directions

We compared the differences in the transition probability between the two transition directions (i.e. States 1 to 2 or States 2 to 1) using the following model:


\begin{eqnarray*}
\textit{transition}\,\textit{probability}\sim{\mathrm{\ }}\textit{transition}{\mathrm{\ }}\textit{direction} + \left( {1{\mathrm{|}}\textit{subject}{\mathrm{\ }}ID} \right)\\
+ \left( {1{\mathrm{|}}sex} \right) + \left( {1{\mathrm{|}}\textit{content}{\mathrm{\ }}\textit{types}} \right).
\end{eqnarray*}

A permutation test (*N* = 10 000) was performed to determine the significance of the mixed-effects model.

#### Differences in the temporal properties between sexes or content types

We compared the differences in the occurrence rate between different conditions (sexes or content types) using the following model:


\begin{eqnarray*}
\textit{occurrence}\,\textit{rate}\sim\textit{condition} + \left( {1{\mathrm{|}}\textit{subject}{\mathrm{\ }}ID} \right).
\end{eqnarray*}

We performed a permutation test (*N* = 10 000) to assess the significance of the mixed-effects model. Similarly, the differences in mean dwell time (or transition probability) between sexes or content types were assessed using the same approach.

#### Differences in laterality fluctuation between sexes or content types

We compared the differences in laterality fluctuation (LF) between different conditions (sexes or content types) using the following model:


\begin{eqnarray*}
\textit{laterality}{\mathrm{\ }}\textit{fluctuations}{\mathrm{\ }}\sim\textit{condition} + \left( {1{\mathrm{|}}\textit{Subject}{\mathrm{\ }}ID} \right).
\end{eqnarray*}

The linear mixed-effects model was fitted for the LF value of each brain region. *P*-values were corrected for multiple comparisons using Benjamini–Hochberg false discovery rate (BH-FDR) corrections (*q*  < 0.05) (Benjamini and Hochberg, 1995).

### Gene expression analysis of dynamic lateralization states

#### Gene expression data

Regional microarray expression data were obtained from six postmortem brains (one female, ages 24.0–57.0 years, mean age 42.50 ± 13.38 years) provided by the Allen Human Brain Atlas (AHBA, https://human.brain-map.org) (Hawrylycz *et al*., 2012). The data were processed using the abagen toolbox (v.0.1.3; https://github.com/rmarkello/abagen) and the HCPex atlas, a 426-region volumetric atlas in MNI space.

First, the microarray probes were annotated using the data provided by Arnatkevic̆iūtė *et al*. (2019). The probes that did not match a valid Entrez ID were discarded. Next, the probes were filtered based on their expression intensity relative to background noise (Quackenbush, 2002). Probes with intensities lower than the background in ≥50% of samples across donors were discarded. In cases where multiple probes matched the expression of the same gene, we selected the probe with the most consistent pattern according to regional variations across donors (i.e. differential stability) (Hawrylycz *et al*., 2015). The differential stability was calculated using the formula:


\begin{eqnarray*}
{{\Delta }_S}\left( p \right) = \frac{1}{{\left( {\begin{array}{@{}*{1}{c}@{}} N\\ 2 \end{array}} \right)}}\mathop \sum \limits_{i = 1}^{N - 1} \mathop \sum \limits_{j = i + 1}^N \rho \left[ {{{B}_i}\left( p \right),{{B}_j}\left( p \right)} \right],
\end{eqnarray*}

where ρ is the Spearman’s rank correlation of the expression of a single probe, *p*, across regions in two donors *B_i_* and *B_j_*, and *N* is the total number of donors. The regions corresponded to the structural designations provided in the ontology from the AHBA.

The MNI coordinates of the tissue samples were updated by performing nonlinear registration using advanced normalization tools (ANTs; https://github.com/chrisfilo/alleninf). Samples were assigned to brain regions in the provided atlas if their MNI coordinates were within 2 mm of a given parcel. To minimize assignment errors, sample-to-region matching was constrained by hemisphere and gross structural divisions (i.e. cortex, subcortex/brainstem, and cerebellum). For example, a sample in the left cortex could be assigned only to an atlas parcel in the left cortex (Arnatkevic̆iūtė *et al*., 2019). If a brain region was not assigned a sample from any donor based on the above procedure, the tissue sample closest to the centroid of that parcel was identified independently for each donor. The average of these samples, weighted by the distance between the parcel centroid and the sample, was calculated across donors to estimate the parcellated expression values for the missing region. Tissue samples that were not assigned to a brain region in the provided atlas were discarded.

To address intersubject variation, tissue sample expression values were normalized across genes using a robust sigmoid function (Fulcher *et al*., 2013):


\begin{eqnarray*}
{{x}_{\textit{norm}}} = \frac{1}{{1 + {\mathrm{exp}}\left( { - \frac{{\left( {x - \langle x\rangle } \right)}}{{{\mathrm{IQ}}{{{\mathrm{R}}}_x}}}} \right)}},
\end{eqnarray*}

where 〈*x*〉 is the median and IQR_*x*_ is the normalized interquartile range of the expression of a single tissue ample across genes. Normalized expression values were then rescaled to the unit interval:


\begin{eqnarray*}
{{x}_{\textit{scaled}}} = \frac{{{{x}_{\textit{norm}}} - min( {{{x}_{\textit{norm}}}} )}}{{max( {{{x}_{\textit{norm}}}} ) - min( {{{x}_{\textit{norm}}}} )}}.
\end{eqnarray*}

The gene expression values were normalized across tissue samples using the same procedure. Samples assigned to the same brain region were averaged separately for each donor and then across donors. The fully pre-processed gene data included a 426 × 15 632 matrix of microarray gene expression data.

#### Identification of genes correlated with dynamic lateralization states

We performed correlation analyses between each group-averaged dynamic lateralization state and the Allen Human Brain spatial gene expression patterns. For each dynamic lateralization state, we calculated the Pearson correlation between each 426-region gene expression vector and the 426-region dynamic lateralization state vector. Multiple comparisons were corrected using BH-FDR corrections (*q* < 0.05). Genes that exhibited significant correlations with lateralization states were identified as dynamic lateralization-related genes.

#### Gene set enrichment analysis

To provide further biological insights into the genes that exhibited significant correlations with the lateralization states, we conducted gene set enrichment analysis (GSEA) with FUMA, an integrative web-based platform (Watanabe *et al*., 2017). We employed the GENE2FUNC function to evaluate the enrichment of the dynamic lateralization-related genes. In the enrichment analysis, hypergeometric tests were performed to determine if the mapped genes were overrepresented in any of the predefined gene sets. Gene sets were obtained from the genes reported in the Genome-Wide Association Study (GWAS) Catalogue (MacArthur *et al*., 2017). Multiple test correction was conducted using BH-FDR corrections with an adjusted *P*-value cutoff of 0.05 and a minimum of two overlapping genes.

### Comparison of dynamic lateralization states identified during the resting state and the processing of Chinese natural speech stimuli

The dynamic brain lateralization was analysed using resting-state fMRI data collected from a sample of 991 participants enrolled in the HCP cohort. Detailed algorithm parameters can be found in a previous study (Wu *et al*., 2022). We quantified the similarity between the group-averaged dynamic lateralization states observed during the Chinese natural listening task and those observed during the resting state. Specifically, we computed the Pearson correlation between the group-averaged centroid maps of the task-based and resting-state dynamic lateralization states to quantify spatial correspondence.

Furthermore, we statistically analysed the temporal properties of the dynamic lateralization states identified during the resting state, including the occurrence rate and mean dwell time. To investigate potential disparities in these temporal properties between sexes, we employed the following linear mixed-effects model, with age, education level, race, and grey matter volume (GMV) as covariates and family ID as a random effect:


\begin{eqnarray*}
occurrence\ \textit{rate}/mean\ \textit{dwell}\ \textit{time} &\sim& sex + age\ + \ \textit{education}\\ &&+ \, \textit{race}\ + GMV + \ (1).
\end{eqnarray*}

## Results

### Categories of the Chinese natural speech stimuli

Participants listened to a set of 20 8- to 12-min narratives covering various genres, including fairy tales, fables, novels, essays, news, and scientific essays. We utilized natural language processing techniques and hierarchical clustering analysis approaches to classify the Chinese natural speech stimuli based on their semantic information. The clustering results were visualized using a dendrogram, revealing two distinct narrative categories (Fig. S1a). Then, we performed an assessment to confirm the optimal number of clusters based on the silhouette criterion (Rousseeuw, 1987). This evaluation affirmed the suitability of classifying narratives into two categories, as indicated by the highest silhouette value (Fig. S1b). Subsequently, we manually labelled one category as narratives with an emotional narrative tone (12 narratives) and the other as narratives with a rational narrative tone (eight narratives) (see Table 1 for details). These labels were used to investigate the influence of the type of contents on brain dynamic lateralization patterns.

**Table 1:** Two categories of the Chinese narrative stimuli.

No.	Title	Genre	Category
1	The Rabbit Who Went to the Moon	Fairy tale	Emotional
2	Severing the Friendship	Fable	Emotional
3	The Family	Novel	Emotional
4	The Class Adviser	Novel	Emotional
5	The Color of Light	Essay	Emotional
6	The Hometown Banyan Trees	Essay	Emotional
7	Police Arrest 26 Suspects After Airing of ‘Crazy Personal Information Black Market’	News	Rational
8	Huabei Connects to the Central Bank’s Credit System, Gradually Covering All User Groups	News	Rational
9	Petroleum Resources	Scientific explanatory essay	Rational
10	Bombed by Negative News, Here’s How to Maintain Mental Health	News	Rational
11	School in the Clouds	Fairy tale	Emotional
12	The Battle of the Yellow Emperor Against Chi You	Fable	Emotional
13	Chronicle of a Blood Merchant	Novel	Emotional
14	Ordinary World	Novel	Emotional
15	Poetic Night Rain	Essay	Emotional
16	The Five Key Flavors of Chinese Food	Essay	Emotional
17	Safeguarding the Vital Thread of Ethnic Unity	News	Rational
18	Chinese President Xi Jinping Empowers Entrepreneurs for Greater Economic Impact	News	Rational
19	Flowing Water on Mars	Scientific explanatory essay	Rational
20	How Does the Brain Perceive the Passage of Time?	Scientific explanatory essay	Rational

### Two distinct dynamic lateralization states associated with rational and emotional contents processing

**Figure 2:**
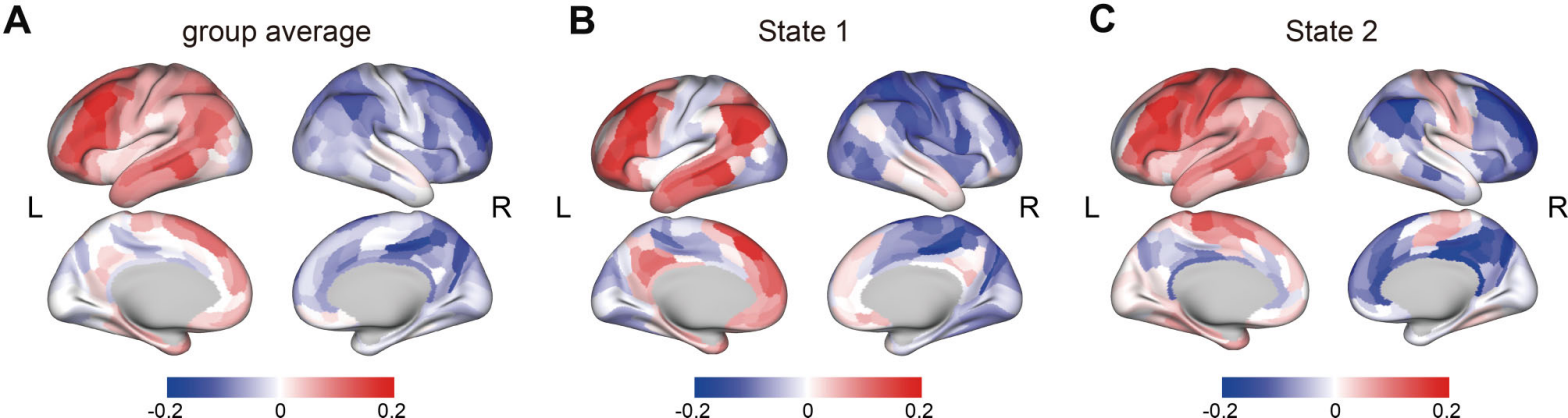
Identification of two dynamic lateralization states. (**A**) The group-averaged lateralization maps across all time windows, with the majority of brain regions in both hemispheres consistently showing lateralization towards the corresponding side. (**B**) State 1 of the two dynamic lateralization states, where the left hemisphere exhibited strong left lateralization in regions such as the dorsolateral prefrontal cortex and inferior parietal cortex (e.g. areas IFSa, p47r, and 45, as defined in the HCPex atlas). (**C**) State 2 of the two dynamic lateralization states, with the right hemisphere displaying pronounced right lateralization in the dorsolateral prefrontal cortex, posterior cingulate cortex, and inferior parietal cortex. B and C depict a distinct reversal in the lateralization pattern of specific brain regions between State 1 and State 2, including the somatosensory cortex, the paracentral lobule, and the middle cingulate cortex. In State 1, these regions in the left hemisphere exhibited right lateralization, whereas in State 2, these same regions within the right hemisphere displayed left lateralization.

We employed an approach combining a sliding window technique and the global signal-based laterality index to calculate the DLI of the 426 brain regions in the HCPex atlas (Huang *et al*., 2022). The group-averaged laterality maps across all time windows are shown in Fig. 2a. For regions in the left hemisphere, most of them showed left lateralization, except for specific ones in the visual cortex, paracentral lobule, middle cingulate, and posterior cingulate, which showed slight right lateralization. In contrast, nearly all right hemisphere regions displayed right lateralization, except for some auditory association areas (such as the superior temporal gyrus area) exhibiting relatively neutral laterality (Fig. 2A).

By employing a two-stage temporal clustering approach based on the Louvain community detection algorithm (Blondel *et al*., 2008), we identified two recurring whole-brain dynamic lateralization states with distinct brain lateralization patterns (Figs 2B and 2C). In both dynamic lateralization states, most brain regions consistently exhibited lateralization towards the hemisphere they belong to. Specifically, in State 1, the left hemisphere regions such as the dorsolateral prefrontal cortex and inferior frontal cortex (e.g. areas IFSa, p47r, and 45, as defined in the HCPex atlas) exhibited strong left lateralization (Fig. 2B). In State 2, right hemisphere regions such as the dorsolateral prefrontal cortex, posterior cingulate cortex, and inferior parietal cortex displayed pronounced right lateralization (Fig. 2c). However, we observed a distinct reversal of the typical lateralization pattern in certain brain regions in these two states, such as the somatosensory cortex, the paracentral lobule, and the middle cingulate cortex. In State 1, these regions in the left hemisphere exhibited right lateralization, whereas in State 2, these regions in the right hemisphere displayed left lateralization.

To elucidate the properties of these two states, we investigated their potential differences with respect to text sentiment. We segmented all the audio files into sentences using NLP techniques and then manually assigned the sentiment score to each sentence. Specifically, sentences with rational contents were labelled 0, and those with emotional contents were labelled 1. After aligning the time windows of the fMRI data with the audio data, we determined the sentiment score for each time window and averaged the scores for each state. The average sentiment score for State 1 was 0.29, which was significantly lower than that for State 2, with an average score of 0.32 (*t* = −3.3, *P* = 9.6 × 10^−4^). According to the predefined criteria for sentiment labelling of the audio data, we classified State 1 as the dynamic lateralization state associated with the processing of rational contents, while State 2 was characterized as the dynamic lateralization state associated with the processing of emotional contents.

### Spatial organization and cognitive functions of the two dynamic lateralization states

**Figure 3:**
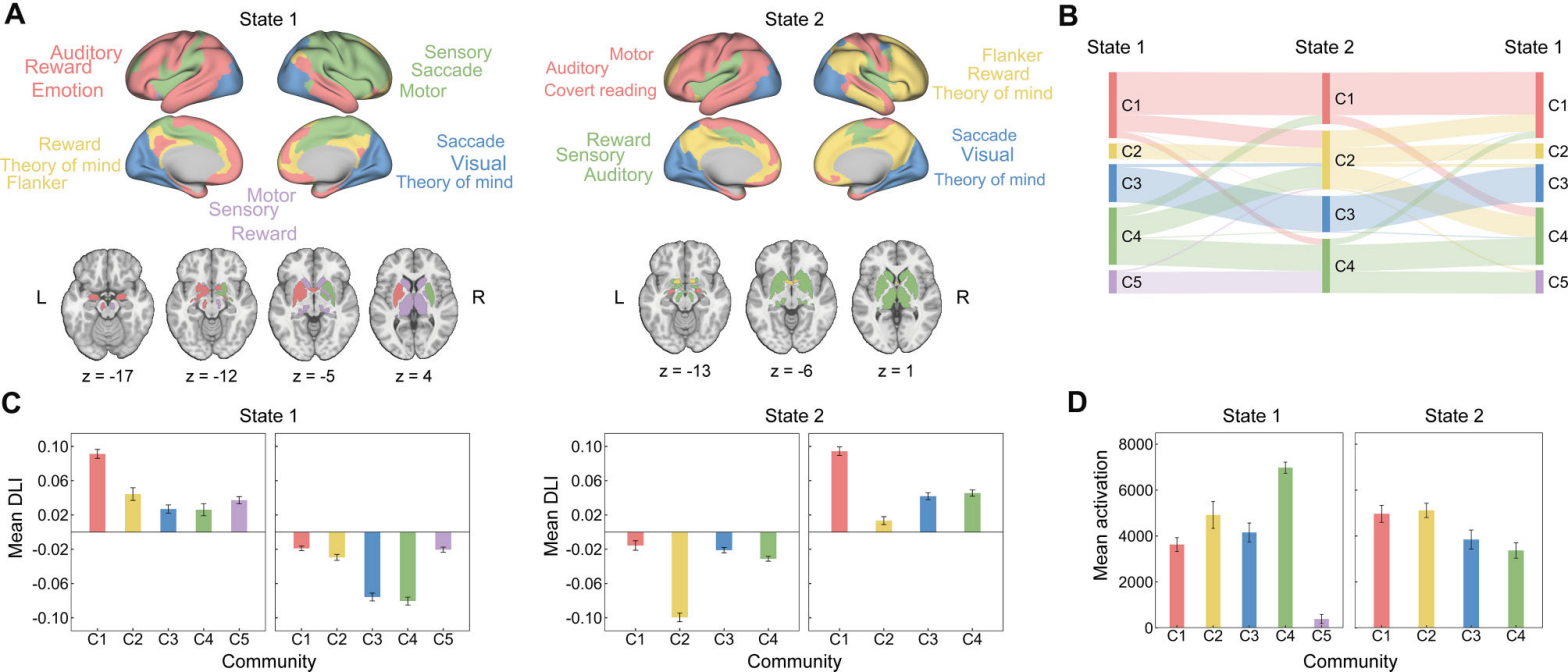
Spatial organization of dynamic lateralization states. (**A**) The community structures within the two dynamic lateralization states. State 1 exhibits the presence of five distinct spatial communities, and State 2 presents four spatial communities. Community 1 (C1)-Language, C2-TOM, C3-Visual, C4-SM, and C5-Subcortical. (**B**) The segmentation and integration of the communities during the state transitions. (**C**) Mean DLI values for communities in the two states. Within each state, the left and right bar plots represent the mean DLI of the left (average of positive values) and right (average of negative values) lateralization in each community, respectively. (**D**) Mean activation values for the communities in these two states. The error bars in C and D denote the mean ± SEM.

To investigate the spatial organization of the two dynamic lateralization states, we conducted a spatial community analysis for each state using the Louvain community detection algorithm (Blondel *et al*., 2008) (Fig. 3A). State 1 exhibited five distinct spatial communities, while State 2 displayed four. To ensure cross-state comparability among these communities, we aligned them by reassigning labels based on their anatomical locations.

To elucidate the cognitive functions associated with the spatial organization of these two states, we employed a predefined cognitive component template (Yeo *et al*., 2015) to characterize the cognitive functions associated with each community. In both states, Community 1 (C1), primarily associated with regions for language processing, was named C1-Language. C2 represented a cluster of regions responsible for higher-level cognitive functions, such as theory of mind, flanker tasks, and reward processing, which was named C2-TOM. C3 was related to lower-level functions such as visual processing and saccades, which was named C3-Visual. C4 was associated with sensory and motor processing, which was named C4-SM. C5 was only observed in State 1 and primarily located within subcortical regions involved in sensory and motor processing, which was named C5-Subcortical.

After aligning the communities in the two states, we employed a CTR matrix to assess how communities are segmented and integrated during state transitions. Overall, C1-Language and C4-SM occupied the large proportions of brain regions in State 1. However, in State 2, the proportions of brain regions distributed among the four communities were relatively balanced (Fig. 3B). When transitioning from State 1 to State 2, C1-Language and C4-SM in State 1 were segmented and subsequently integrated into other communities in State 2. Specifically, C2-TOM in State 2 included all of C2-TOM, 37% of C4-SM, 26% of C1-Language, 9% of C3-Visual, and 8% of C5-Subcortical from State 1. C4-SM in State 2 included half of the brain regions from C4-SM in State 1 and nearly all of C5-Subcortical from State 1. On the other hand, C3-Visual remained relatively stable during the state transition, with most brain regions remaining within their respective communities. Conversely, during the transition from State 2 to State 1, the communities underwent reverse segmentation and integration processes.

Mean DLI values for the communities in these two states are displayed in Fig. 3C, showing a significant contrast between the two states. In State 1, the C1-Language and C2-TOM clusters showed left-lateralization. While C1-Language was consistently left-lateralized, C2-TOM showed significant right-lateralization in State 2. Notably, C3-Visual and C4-SM, associated with visual, sensory, and motor processing, was predominantly localized to the right hemisphere in State 1 but displayed a slightly left-forward lateralization in State 2. These shifts in hemispheres may be related to the higher prevalence of emotion processing in State 2.

Moreover, we computed the mean activation values for the communities in both states to estimate the functional involvements (Fig. 3D). In State 1, the activation values of the five communities varied considerably, with significant differences in activation observed between every pair of communities after Bonferroni correction (*P* < 0.05/10). Specifically, in State 1, C4-SM exhibited the highest activation, followed by C2-TOM. In State 2, the activation values of the four communities were relatively balanced, yet significant differences in activation were still observed after Bonferroni correction (*P* < 0.05/6). C1-Language and C2-TOM, which were associated with higher-level functions, displayed greater activation values than C3-Visual and C4-SM, which were involved in lower-level functioning.

### Differences in the temporal properties of dynamic lateralization states and influences of sexes and content types

**Figure 4:**
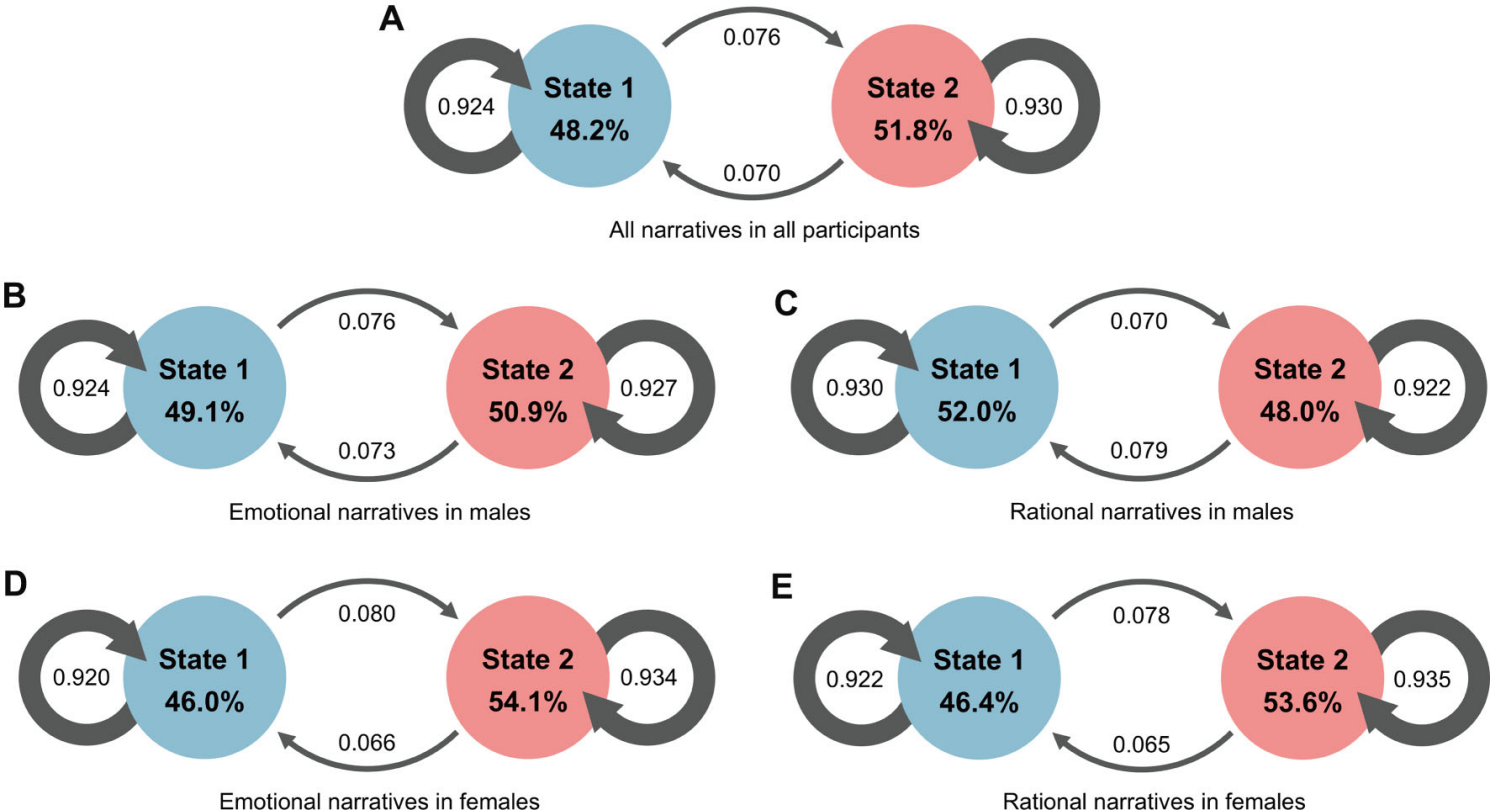
Temporal properties of dynamic lateralization states. (**A**) Occurrence rates and the mean dwell times of the two states, as well as the transition probabilities between states across all participants and all narrative sessions. The thickness of inward arrows represents the mean dwell time in each state, and the numeric labels placed near the arrows indicate the transition probabilities along each direction. For example, in A, the probabilities correspond to transitions from State 1 to itself (0.924), from State 1 to State 2 (0.076), from State 2 to State 1 (0.070), and from State 2 to itself (0.930). (**B–E**) These temporal properties for different sexes and content types. Linear mixed-effects models were employed to examine their potential effects on these temporal properties, with age and the number of time windows as covariates. The significance of the mixed-effects models was assessed by permutation tests (*N* = 10 000).

After the exploration of spatial properties, we further examined the disparities in the temporal properties of the two lateralization states. We employed a linear mixed-effects model to calculate the differences in these two states in the occurrence rate, mean dwell time, transition probability, and LF. The temporal properties of the two dynamic laterality states are summarized in Fig. 4. Specifically, Fig. 4A shows the overall transition dynamics between the two states, where the thickness of inward arrows represents the mean dwell time and the numeric labels denote normalized transition probabilities across participants. Figure 4B further illustrates the direction of transitions and their corresponding probabilities, while Fig. 4C–E display these temporal properties separately by sex and content type. The influences of both sex and narrative content (emotional vs. rational) on these temporal properties were then estimated using linear mixed-effects models.

First, we found that State 2 occurs more than State 1 with significantly higher occurrence rate (mean of State 1 = 48.2%, *n* = 400; mean of State 2 = 51.8%, *n* = 400; *P* = 1.9 × 10^−3^) (Fig. 4A). Moreover, sex and content had considerable impacts on the occurrence rates in both states. Specifically, State 1 happens more in males than in females, with significant differences in occurrence rates (mean of males = 50.2%, *n* = 200; mean of females = 46.1%, *n* = 200; *P* = 6.0 × 10^−3^). Further analyses revealed that this disparity primarily occurred when participants were presented with narratives with rational contents (mean of males = 52.0%, mean of females = 46.4%, *P* = 0.02) (Fig. 4C, E). Meanwhile, when participants were presented with narratives with emotional contents, the sex differences disappeared. Conversely, State 2 occurs more in females than in males (mean of males = 49.8%, *n* = 200; mean of females = 53.9%, *n* = 200; *P* = 6.0 × 10^−3^), and this difference was primarily associated with the presentation of rational narratives (mean of males = 48.0%, mean of females = 53.6%, *P* = 0.02) (Fig. 4C, E). Additionally, no significant difference in the occurrence rate was observed between content types for either state.

Furthermore, the mean dwell time was significantly longer in State 2 than in State 1 (mean of State 1 = 15.6, *n* = 400; mean of State 2 = 16.9, *n* = 400; *P* = 0.02) (Fig. 4A). This difference was particularly pronounced in females (emotional narratives: mean of State 1 = 14.2, mean of State 2 = 17.0, *P* = 1.0 × 10^−4^; rational narratives: mean of State 1 = 15.5, mean of State 2 = 18.2, *P* = 8.4 × 10^−3^) (Fig. 4D, E). Furthermore, sex differences were observed only in State 1 with males dwelling longer than females (mean of males = 16.4, *n* = 200; mean of females = 14.7, *n* = 200; *P* = 0.02). No significant differences in mean dwell time were observed between content types for either state.

Additionally, we explored the differences in transition probability. We found that the probability from States 1 transferred to 2 was significantly higher than that from States 2 to 1 (mean of States 1 to 2 transition = 0.076, *n* = 400; mean of States 2 to 1 transition = 0.070, *n* = 400; *P* = 0.01) (Fig. 4A). After dividing the population according to sex, this difference was observed only in females during the processing of both emotional narratives (mean of States 1 to 2 transition = 0.080, mean of States 2 to 1 transition = 0.066, *P* = 6.0 × 10^−4^) and rational narratives (mean of States 1 to 2 transition = 0.078, mean of States 2 to 1 transition = 0.065, *P* = 0.01) (Fig. 4D, E). No differences in transition probabilities were observed in males. Furthermore, sex differences were found in the transition probability from States 2 to 1, with the transition probability significantly lower in females than in males (mean transition probability of females = 0.066, *n* = 200; mean transition probability of males = 0.075, *n* = 200; *P* = 2.1 × 10^−3^). This difference was observed when participants were presented with either emotional (mean transition probability of females = 0.066, mean transition probability of males = 0.073, *P* = 0.04) or rational narratives (mean transition probability of females = 0.065, mean transition probability of males = 0.079, *P* = 0.01). No sex differences were found in the transition probability from States 1 to 2. Additionally, no significant differences in transition probabilities were observed between content types.

**Figure 5:**
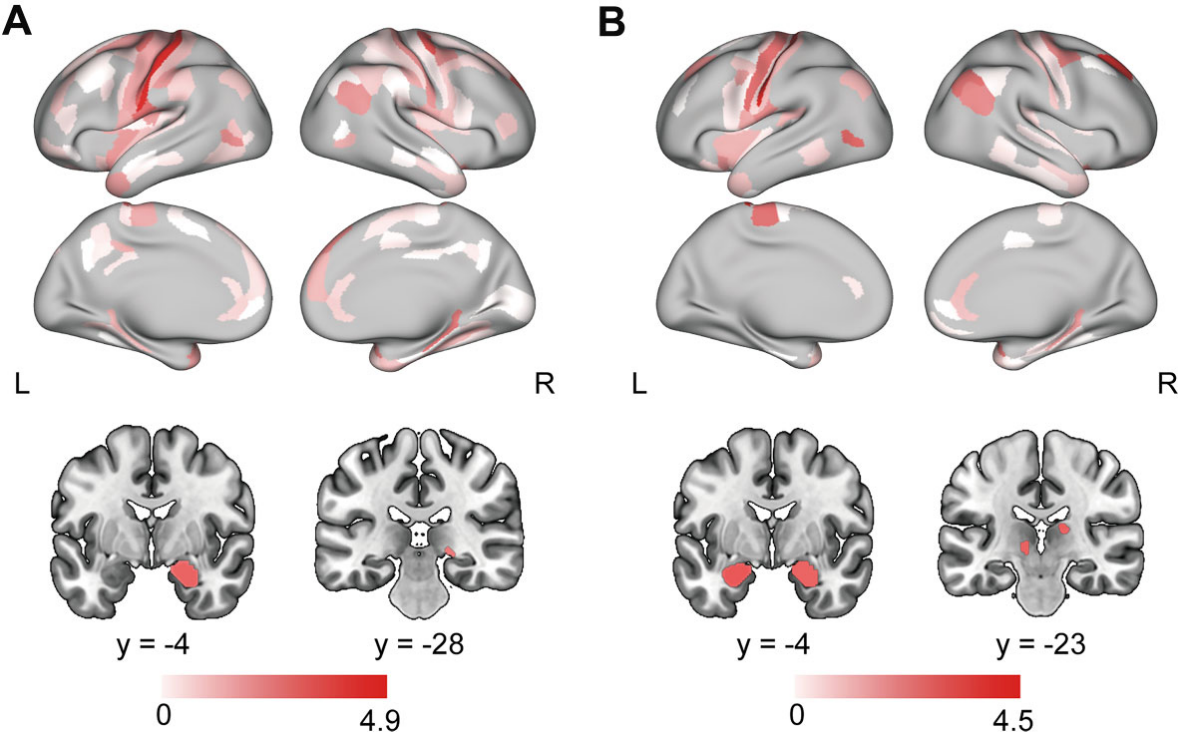
Differences in the laterality fluctuation between sexes (males > females) during the processing of narratives with rational contents. (**A, B**) Brain regions exhibiting significant differences in laterality fluctuation in State 1 and State 2, respectively. The statistical analyses were conducted using linear mixed-effects models, with age and the number of time windows as covariates. The linear mixed-effects model was fitted according to the laterality fluctuation (LF) of each brain region using BH-FDR corrections for multiple comparisons (*q*  < 0.05).

Significant sex differences in regional LF were particularly evident during the processing of rational narratives with males generally exhibited higher LF values. In State 1, these differences were primarily observed in specific brain regions, including the somatosensory and motor regions, medial prefrontal cortex, posterior cingulate cortex, parahippocampus, insular, and amygdala (Fig. 5A). In State 2, the brain regions exhibiting significant differences in LF were similar to those in State 1, primarily involving the somatosensory and motor regions, anterior insular cortex, amygdala, dorsolateral prefrontal cortex, and inferior parietal cortex (Fig. 5B). Notably, only one region in the insular cortex in the right hemisphere (i.e. area PoI1) showed significant sex differences in LF during the processing of emotional narratives (Fig. S2).

### Gene set enrichments for dynamic lateralization states

To explore the genetic underpinnings of the two dynamic lateralization states, we conducted a GSEA of the two states. Correlation analyses between gene expression and dynamic lateralization states showed significant associations with State 1 and State 2, and we identified 4080 and 3355 genes with significant correlations in States 1 and 2, respectively. These identified genes were associated with specific mental disorders, cognitive functions, and hormones, particularly sex hormones, based on the gene sets from the GWAS Catalogue (MacArthur *et al*., 2017).

**Figure 6:**
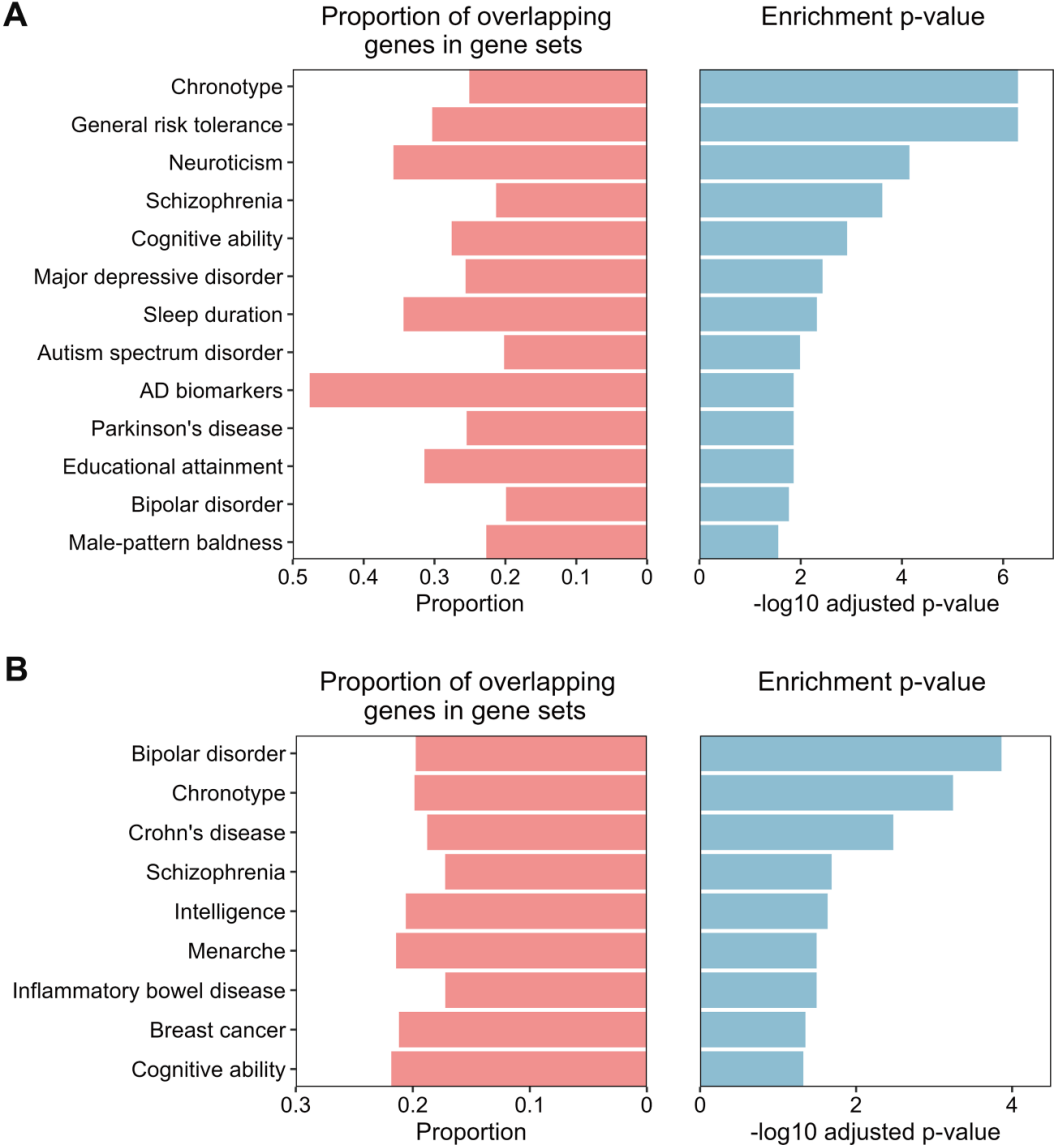
Gene set enrichment analysis for genes associated with dynamic lateralization states. (**A, B**) The biological functions associated with the genes related to the two dynamic lateralization states, as retrieved from the Genome-Wide Association Study (GWAS) Catalogue. Multiple test correction was conducted using BH-FDR corrections, with an adjusted *P*-value cutoff of 0.05 and a minimum of two overlapping genes.

More specifically, significant enrichment of genes associated with specific mental disorders, such as neuroticism (*q*  = 7.1 × 10^−5^), schizophrenia (*q*  = 2.4 × 10^−4^), and autism spectrum disorder (*q*  = 0.01), was observed in State 1. Furthermore, significant enrichment of genes associated with specific cognitive functions, including general risk tolerance (*q*  = 5.1 × 10^−7^), cognitive ability (*q*  = 1.2 × 10^−3^), and educational attainment (*q*  = 0.01), was observed in State 1. Intriguingly, significant enrichment of genes related to hormones, notably male sex hormones, encompassing traits such as chronotype (*q*  = 5.1 × 10^−7^), sleep duration (*q*  = 4.8 × 10^−3^), and male-pattern baldness (*q*  = 0.03), was also observed in State 1 (Fig. 6a).

In contrast, different gene enrichment patterns were observed in State 2 than in State 1. In addition to genes associated with mental disorders (e.g. bipolar disorder, *q* = 1.4 × 10^−4^) and cognitive functions (e.g. intelligence, *q* = 0.02), enrichment of genes associated with inflammatory bowel diseases, specifically Crohn’s disease (*q* = 3.3 × 10^−3^), was observed in State 2. Interestingly, significant enrichment of genes associated with female sex hormones, including menarche (*q* = 0.03) and breast cancer (*q*  = 0.04), was also observed in State 2 (Fig. 6B).

### Similarities in dynamic lateralization states between the resting state and Chinese natural speech task

Finally, we investigates whether the dynamic lateralization states identified during the Chinese natural listening task represent inherent lateralization patterns during resting state within the human brain. Here, we conducted an analysis of dynamic brain lateralization using resting-state fMRI data collected from a sample of 991 participants enrolled in the HCP cohort (mean age 28.7 ± 3.7 years, 528 females).

Three distinct dynamic lateralization states were identified during the resting state, as shown in Fig. S3. Interestingly, two of these states exhibited significant similarities with the two dynamic lateralization states identified during the processing of Chinese natural speech stimuli (for State 1, *r* = 0.79, *P* = 6.4 × 10^−79^; for State 2, *r* = 0.79, *P* = 5.3 × 10^−79^). This implies that the two dynamic lateralization states observed during the processing of Chinese natural speech stimuli reflect the inherent lateralization patterns within the human brain. These states, characterized by left-lateralized and right-lateralized regions predominantly engaged in higher-level cognitive processes, exhibited heightened activity during the Chinese natural listening task.

Furthermore, the statistical analyses using linear mixed-effects models revealed similar sex differences in temporal properties of these two states. We found that the state, which is similar to State 2 identified during the Chinese natural speech task, occurs significantly more in females than in males (for occurrence rate, *t* = 3.0, *P* = 2.5 × 10^−3^; for mean dwell time, *t* = 3.3, *P* = 1.2 × 10^−3^; 528 females and 463 males). This suggests that sex influences on the temporal properties of the dynamic lateralization states are also inherent, which are similar in both the resting state and during the processing of Chinese natural speech stimuli.

### Validation analysis

To rule out the potential impacts of varying time window lengths, we assessed the reproducibility of the DLI findings using two additional time window lengths, 18 TR and 25 TR. We consistently identified two distinct dynamic lateralization states (Fig. S4). Moreover, the two states identified using 18-TR and 25-TR time windows were both remarkably similar to those identified with the 20-TR time window (18 TR: for State 1, *r* = 0.99, *P* < 1.0 × 10^−15^; for State 2, *r* = 0.99, *P* < 1.0 × 10^−15^; 25 TR for State 1, *r* = 0.99, *P* < 1.0 × 10^−15^; for State 2, *r* = 0.99, *P* < 1.0 × 10^−15^). These results demonstrate the robustness of the DLI analysis, confirming that our results can be replicated regardless of the chosen time window length.

## Discussion

In this study, we investigated the dynamic nature of whole-brain lateralization during Chinese language processing and explored the potential influences of sex and content (emotional or rational contents), as well as the genetic underpinnings of the observed dynamic lateralization. Remarkably, our findings revealed the existence of two distinct dynamic lateralization states. While the language system was predominantly found in the left hemisphere, intriguingly, we observed converse lateralization patterns in high-level functioning systems. These two states, associated with higher-level functioning systems exhibiting left- or right-lateralization, corresponded to the processing of rational and emotional contents, respectively. More interestingly, males displayed a pronounced inclination for the former state, while females consistently exhibited a preference for the latter state, especially during the processing of rational contents. Furthermore, our investigation revealed genetic evidence that supports these observed sex differences in language processing.

We identified two dynamic states with switched hemispheric specialization, which reflects a core property of human cognition and a marker of efficient functioning. Our study revealed that brain regions associated with language processing, such as covert and overt reading, were consistently located in the left hemisphere for both dynamic lateralization states. This emphasized the centralized role of the left hemisphere for basic language functions, which would benefit the rapid processing (Budisavljevic *et al*., 2015; Braga *et al*., 2020; Olulade *et al*., 2020). Moreover, the most notable distinction between these two states is the lateralization patterns of higher-level cognitive systems that assist in language processing in real-life scenarios. Specifically, in State 1, which was strongly associated with the processing of rational contents, brain regions associated with higher-level functions such as reward processing, theory of mind, and emotion showed left lateralization. Conversely, in State 2, which was associated with the processing of emotional contents, the brain regions involved in higher-level cognitive processes such as reward processing, theory of mind, and flanker tasks exhibited right lateralization. This phenomenon showed the coordinated efforts of higher-level cognitive streams between brain hemispheres, which would be superimposed on the basic language processing during language comprehension to enhance cognitive capacity and fluency (Jung-Beeman *et al*., 2004; Fernandino *et al*., 2016). It could be inferred that, in real life, the realization of efficiently language processing is not limited to one single system, but requires the dynamic and precise cooperation of multiple systems across the whole brain (Labache *et al*., 2023). The specialized processing for each hemisphere also echoed prior studies suggesting that the left hemisphere is predominantly related to mathematical and logical reasoning (Westerhausen *et al*., 2014; Friederici, 2017; Verhelst*et al*., 2021), while the right hemisphere is related to emotion processing (Stoyanov *et al*.,Nikolova, 2012; Watanabe *et al*., 2015).

Additionally, we observed the noteworthy sex differences in language processing, and these disparities were influenced by the type of contents. Previous research on sex differences in brain lateralization during language processing revealed the tendency for more left-hemisphere engagement in males (Burman *et al*., 2013; Clements *et al*., 2006; Packheiser *et al*., 2020; Xu *et al*., 2020; Yang *et al*., 2020). Here, we revealed a more detailed description about the sex differences in language processing. Irrespective of the content type, females consistently exhibited a preference for State 2, characterized by higher-level functions with more right lateralization, as evidenced by significantly higher occurrence rates and a greater inclination to transition towards this state. In contrast, males displayed a pronounced inclination for State 1, characterized by higher-level functions with more left lateralization, particularly during the processing of rational contents. Our findings indicate that during real-life language processing, females tend to employ a perceptual thinking approach, while males tend to employ rational thinking styles. Notably, this intriguing inference provides the neural mechanism explanation for the empathizing–systemizing theory, which suggests that females typically demonstrate greater empathy, while males exhibit a preference for systems-oriented approaches (Baron-Cohen *et al*., 2003; Greenberg *et al*., 2018, 2023).

The GSEAs based on the two dynamic lateralization states reflect the fundamental questions about the genetic origin and sex differences of language capacities in cognitive neuroscience. A prominent distinction between these states was observed: in State 1, genes associated with male sex hormones, such as male-pattern baldness, were enriched, whereas in State 2, genes associated with female sex hormones, such as menarche and breast cancer, were enriched. This finding showed how the sex hormones would influence the language dynamic lateralization, which are consistent with the findings that males were more inclined towards State 1, while females consistently preferred State 2. Interestingly, prior research has reported the substantial impact of sex hormones on language development (Bowers *et al*., 2013; Enard *et al*., 2002; Haesler *et al*., 2007; Wermke *et al*., 2018). Additionally, previous studies have indicated the role of biological sex and sex hormones in shaping functional cerebral asymmetries, including language lateralization (Hausmann, 2017; Maney, 2016). By comparing dynamic states during resting-state with those during natural language processing, our findings clarify the origin of the two dynamic lateralization states observed during language tasks. We demonstrate that these states do not arise *de novo* but instead reflect inherent lateralization patterns present in the human brain, which become heightened under language processing demands. These results strongly support the view that task-evoked brain activity is an extension of its intrinsic dynamic organization. The presence of language-like lateralization patterns during rest, acting as a ‘functional blueprint’ for task-related processes, indicates that the resting brain is far from a neutral baseline. Rather, it represents an active, organized regime of cognitive preparedness. The reuse of such pre-configured networks constitutes a metabolically and computationally efficient mechanism, enabling the brain to rapidly adapt to environmental demands by selectively stabilizing relevant intrinsic states instead of generating new ones *de novo*. Moreover, consistent sex differences observed in both resting and task conditions suggest that hemispheric preference is an intrinsic trait influenced by sex. This framework offers a potential explanation for sex differences observed in other cognitive and behavioural domains, suggesting they may stem from inherent differences in dynamic brain organization.

However, it is important to acknowledge some limitations of our study. First, subject to the fact that the scanning time for each participant was very long, this study only included 20 participants. However, each participant was acquiring 20 scans to simulate different language contents that occur in daily life, which would increase the effective sample size. Additionally, we made efforts to achieve sex balance by recruiting 10 female participants. Future studies with larger and more diverse samples would validate our findings. Second, while the emotional/rational dichotomy for text materials is rooted in common NLP practice, it may not fully capture the multidimensional cognitive processes engaged by language. Future research would benefit from developing a more fine-grained, cognitively inspired annotation framework to more comprehensively explore the language–brain relationship. Another limitation is that the individuals in our study were all young people with high-level education backgrounds (mean age 23.7 ± 1.8 years). It would be valuable to investigate the dynamic lateralization states during Chinese language processing in participants of different ages, including children and older adults. This would allow us to explore potential age-related changes in lateralization patterns and their implications for language processing. Furthermore, our analyses were specifically based on the Chinese language. It would be interesting and important to investigate dynamic lateralization states during English language or other language processing to examine the generalizability of our findings across different linguistic contents.

In conclusion, our study provides valuable insights into the dynamic nature of brain lateralization during Chinese language processing. The identification of two distinct lateralization states improves our understanding of interhemispheric coordination during language processing, emphasizing the dynamic shift in higher-level cognitive functions between left and right lateralization patterns. Furthermore, the associations of these lateralization states with sex differences and the processed contents (emotional or rational contents) offer insights into sex-specific patterns in language processing. Moreover, our genetic-level analyses suggest that the observed sex differences in language lateralization may be influenced by sex hormones. Future research should focus on replicating and expanding our findings utilizing larger and more diverse populations, including individuals with different ages and/or neurodevelopmental disorder, as well as investigating the cognitive significance of the observed dynamic lateralization states. As such, understanding the interactions linking the biological underpinnings of dynamic lateralization, cognition and development would have significant implications for both cognitive neuroscience and developmental biology.

## Supplementary Material

kkag003_Supplemental_File

## Data Availability

The 7T task-based fMRI data utilized in this study were collected by Fudan University. All participants provided fully informed consent, and the research procedures adhered to ethical guidelines approved by the Fudan University Institutional Review Board. Access to the dataset is available upon request by contacting the corresponding author. Python 3.9.7 was utilized for the analysis of audio texts using NLP techniques. The NLP techniques applied in this study included the ‘Jieba’ Chinese text segmentation system (https://github.com/fxsjy/jieba/) and text vectorization (https://github.com/Embedding/Chinese-Word-Vectors). Gene expression data were processed in Python using the abagen toolbox (v.1.3; https://github.com/rmarkello/abagen). MATLAB 2018b was utilized to calculate the whole-brain DLI and perform statistical analyses. The MATLAB Brain Connectivity Toolbox (https://sites.google.com/site/bctnet/) was used to perform community detection based on the whole-brain DLI values to identify the dynamic lateralization states and spatial communities of the states. In addition, the MATLAB functions ‘linkage’ and ‘fitglme’ were utilized for the hierarchical clustering of the vectorized narratives processed through NLP techniques and for the implementation of the linear mixed-effects models, respectively.

## References

[bib1] Arnatkevic̆iūtė A, Fulcher BD, Fornito A (2019) A practical guide to linking brain-wide gene expression and neuroimaging data. Neuroimage. 189:353–67. 30648605 10.1016/j.neuroimage.2019.01.011

[bib2] Baron-Cohen S, Richler J, Bisarya D et al. (2003) The systemizing quotient: an investigation of adults with Asperger syndrome or high–functioning autism, and normal sex differences. Phil Trans R Soc Lond B. 358(1430):361–74. 12639333 10.1098/rstb.2002.1206 PMC1693117

[bib3] Benjamini Y, Hochberg Y (1995) Controlling the false discovery rate: a practical and powerful approach to multiple testing. J R Stat Soc Series B Stat Methodol. 57(1):289–300.

[bib4] Bi YC (2021) Dual coding of knowledge in the human brain. Trends Cogn Sci. 25(10):883–95. 34509366 10.1016/j.tics.2021.07.006

[bib5] Bialystok E, Craik FI, Klein R et al. (2004) Bilingualism, aging, and cognitive control: evidence from the Simon task. Psychol Aging. 19(2):290–303. 15222822 10.1037/0882-7974.19.2.290

[bib6] Binder JR, Frost JA, Hammeke TA et al. (1997) Human brain language areas identified by functional magnetic resonance imaging. J Neurosci. 17(1):353–62. 8987760 10.1523/JNEUROSCI.17-01-00353.1997 PMC6793702

[bib7] Blondel VD, Guillaume J-L, Lambiotte R et al. (2008) Fast unfolding of communities in large networks. J Stat Mech. 2008(10):P10008.

[bib8] Bowers JM, Perez-Pouchoulen M, Edwards NS et al. (2013) Foxp2 mediates sex differences in ultrasonic vocalization by rat pups and directs order of maternal retrieval. J Neurosci. 33(8):3276–83. 23426656 10.1523/JNEUROSCI.0425-12.2013 PMC3727442

[bib9] Braga RM, DiNicola LM, Becker HC et al. (2020) Situating the left-lateralized language network in the broader organization of multiple specialized large-scale distributed networks. J Neurophysiol. 124(5):1415–48. 32965153 10.1152/jn.00753.2019 PMC8356783

[bib10] Budisavljevic S, Dell’Acqua F, Rijsdijk FV et al. (2015) Age-related differences and heritability of the perisylvian language networks. J Neurosci. 35(37):12625–34. 26377454 10.1523/JNEUROSCI.1255-14.2015 PMC4571601

[bib11] Burman DD, Minas T, Bolger DJ et al. (2013) Age, sex, and verbal abilities affect location of linguistic connectivity in ventral visual pathway. Brain Lang. 124(2):184–93. 23376366 10.1016/j.bandl.2012.12.007 PMC3572208

[bib12] Chai LR, Mattar MG, Blank IA et al. (2016) Functional network dynamics of the language system. Cereb Cortex. 26(11):4148–59. 27550868 10.1093/cercor/bhw238 PMC5066829

[bib13] Clements AM, Rimrodt SL, Abel JR et al. (2006) Sex differences in cerebral laterality of language and visuospatial processing. Brain Lang. 98(2):150–8. 16716389 10.1016/j.bandl.2006.04.007

[bib14] Doron KW, Bassett DS, Gazzaniga MS (2012) Dynamic network structure of interhemispheric coordination. Proc Natl Acad Sci USA. 109(46):18661–8. 23112199 10.1073/pnas.1216402109 PMC3503189

[bib15] Enard W, Przeworski M, Fisher SE et al. (2002) Molecular evolution of FOXP2, a gene involved in speech and language. Nature. 418(6900):869–72. 12192408 10.1038/nature01025

[bib16] Esteves M, Lopes SS, Almeida A et al. (2020) Unmasking the relevance of hemispheric asymmetries—Break on through (to the other side). Prog Neurobiol. 192:101823. 32433927 10.1016/j.pneurobio.2020.101823

[bib17] Federmeier KD, Jongman SR, Szewczyk JM (2020) Examining the role of general cognitive skills in language processing: a window into complex cognition. Curr Dir Psychol Sci. 29(6):575–82. 33584021 10.1177/0963721420964095 PMC7877800

[bib18] Fedorenko E, Hsieh P-J, Nieto-Castañón A et al. (2010) New method for fMRI investigations of language: defining ROIs functionally in individual subjects. J Neurophysiol. 104(2):1177–94. 20410363 10.1152/jn.00032.2010 PMC2934923

[bib19] Fedorenko E, Thompson-Schill SL (2014) Reworking the language network. Trends Cogn Sci. 18(3):120–6. 24440115 10.1016/j.tics.2013.12.006 PMC4091770

[bib20] Feng G, Chen H-C, Zhu Z et al. (2015) Dynamic brain architectures in local brain activity and functional network efficiency associate with efficient reading in bilinguals. Neuroimage. 119:103–18. 26095088 10.1016/j.neuroimage.2015.05.100

[bib21] Fernandino L, Binder JR, Desai RH et al. (2016) Concept representation reflects multimodal abstraction: a framework for embodied semantics. Cereb Cortex. 26(5):2018–34. 25750259 10.1093/cercor/bhv020 PMC4830284

[bib22] Friederici AD (2017) Evolution of the neural language network. Psychon Bull Rev. 24(1):41–7. 27368631 10.3758/s13423-016-1090-x PMC5325853

[bib23] Fulcher BD, Little MA, Jones NS (2013) Highly comparative time-series analysis: the empirical structure of time series and their methods. J R Soc Interface. 10(83):20130048. 23554344 10.1098/rsif.2013.0048 PMC3645413

[bib24] Gerrits R, Verhelst H, Vingerhoets G (2020) Mirrored brain organization: statistical anomaly or reversal of hemispheric functional segregation bias?. Proc Natl Acad Sci USA. 117(25):14057–65. 32513702 10.1073/pnas.2002981117 PMC7322015

[bib25] Glasser MF, Coalson TS, Robinson EC et al. (2016) A multi-modal parcellation of human cerebral cortex. Nature. 536(7615):171–8. 27437579 10.1038/nature18933 PMC4990127

[bib26] Greenberg DM, Warrier V, Abu-Akel A et al. (2023) Sex and age differences in “theory of mind” across 57 countries using the English version of the “Reading the Mind in the Eyes” Test. Proc Natl Acad Sci USA. 120(1):e2022385119. 36584298 10.1073/pnas.2022385119 PMC9910622

[bib27] Greenberg DM, Warrier V, Allison C et al. (2018) Testing the empathizing–systemizing theory of sex differences and the extreme male brain theory of autism in half a million people. Proc Natl Acad Sci USA. 115(48):12152–7. 30420503 10.1073/pnas.1811032115 PMC6275492

[bib28] Haesler S, Rochefort C, Georgi B et al. (2007) Incomplete and inaccurate vocal imitation after knockdown of FoxP2 in songbird basal ganglia nucleus Area X. PLoS Biol. 5(12):e321. 18052609 10.1371/journal.pbio.0050321 PMC2100148

[bib29] Hakim AA, Erwin A, Eng KI et al. (2014) Automated document classification for news article in Bahasa Indonesia based on term frequency inverse document frequency (TF-IDF) approach. In: 2014 6th International Conference on Information Technology and Electrical Engineering (ICITEE), Yogyakarta, Indonesia, 1–4.

[bib30] Hausmann M (2017) Why sex hormones matter for neuroscience: a very short review on sex, sex hormones, and functional brain asymmetries. J Neurosci Res. 95(1-2):40–9. 27870404 10.1002/jnr.23857

[bib31] Hawrylycz M, Miller JA, Menon V et al. (2015) Canonical genetic signatures of the adult human brain. Nat Neurosci. 18(12):1832–44. 26571460 10.1038/nn.4171 PMC4700510

[bib32] Hawrylycz MJ, Lein ES, Guillozet-Bongaarts AL et al. (2012) An anatomically comprehensive atlas of the adult human brain transcriptome. Nature. 489(7416):391–9. 22996553 10.1038/nature11405 PMC4243026

[bib33] Hiltunen T, Kantola J, Abou Elseoud A et al. (2014) Infra-slow EEG fluctuations are correlated with resting-state network dynamics in fMRI. J Neurosci. 34(2):356–62. 24403137 10.1523/JNEUROSCI.0276-13.2014 PMC6608153

[bib34] Huang CC, Rolls ET, Feng J et al. (2022) An extended Human Connectome Project multimodal parcellation atlas of the human cortex and subcortical areas. Brain Struct Funct. 227(3):763–78. 34791508 10.1007/s00429-021-02421-6

[bib35] Huth AG, De Heer WA, Griffiths TL et al. (2016) Natural speech reveals the semantic maps that tile human cerebral cortex. Nature. 532(7600):453–8. 27121839 10.1038/nature17637 PMC4852309

[bib36] Joliot M, Tzourio-Mazoyer N, Mazoyer B (2016) Intra-hemispheric intrinsic connectivity asymmetry and its relationships with handedness and language lateralization. Neuropsychologia. 93:437–47. 26988116 10.1016/j.neuropsychologia.2016.03.013

[bib37] Jung-Beeman M, Bowden EM, Haberman J et al. (2004) Neural activity when people solve verbal problems with insight. PLoS Biol. 2(4):e97. 15094802 10.1371/journal.pbio.0020097 PMC387268

[bib38] Kansaku K (2000) Sex differences in lateralization revealed in the posterior language areas. Cereb Cortex. 10(9):866–72. 10982747 10.1093/cercor/10.9.866

[bib39] Kimura D (1992) Cognitive function: sex differences and hormonal influence. Neuroscience year: supplement. 2:41–3.

[bib40] Kucyi A, Tambini A, Sadaghiani S et al. (2018) Spontaneous cognitive processes and the behavioral validation of time-varying brain connectivity. Network Neurosci. 2(4):397–417. 10.1162/netn_a_00037 PMC6195165 30465033

[bib41] Labache L, Ge T, Yeo BT et al. (2023) Language network lateralization is reflected throughout the macroscale functional organization of cortex. Nat Commun. 14(1):3405. 37296118 10.1038/s41467-023-39131-y PMC10256741

[bib42] Landis JR, Koch GG (1977) An application of hierarchical kappa-type statistics in the assessment of majority agreement among multiple observers. Biometrics. 33:363–74. 884196

[bib43] Lazard DS, Collette JL, Perrot X (2012) Speech processing: from peripheral to hemispheric asymmetry of the auditory system. Laryngoscope. 122(1):167–73. 22095864 10.1002/lary.22370

[bib44] Levy J (1969) Possible basis for the evolution of lateral specialization of the human brain. Nature. 224:614–615. 5346604 10.1038/224614a0

[bib45] Li S, Zhao Z, Hu R et al., (2018) Analogical reasoning on Chinese morphological and semantic relations. In: Proceedings of the 56th Annual Meeting of the Association for Computational Linguistics (Volume 2: Short Papers), p. 138–43. Melbourne, Australia: Association for Computational Linguistics.

[bib46] MacArthur J, Bowler E, Cerezo M et al. (2017) The new NHGRI-EBI Catalog of published genome-wide association studies (GWAS Catalog). Nucleic Acids Res. 45(D1):D896–901. 27899670 10.1093/nar/gkw1133 PMC5210590

[bib47] Makuuchi M, Friederici AD (2013) Hierarchical functional connectivity between the core language system and the working memory system. Cortex. 49(9):2416–23. 23480847 10.1016/j.cortex.2013.01.007

[bib48] Maney DL (2016) Perils and pitfalls of reporting sex differences. Phil Trans R Soc B. 371(1688):20150119. 26833839 10.1098/rstb.2015.0119 PMC4785904

[bib49] McAvoy M, Mitra A, Coalson RS et al. (2015) Unmasking language lateralization in human brain intrinsic activity. Cereb Cortex. 26(4):1733–46. 25636911 10.1093/cercor/bhv007 PMC4785953

[bib50] Nielsen F (2016) Introduction to HPC with MPI for Data Science. Cham: Springer.

[bib51] Novick JM, Trueswell JC, Thompson‐Schill SL (2010) Broca’s area and language processing: evidence for the cognitive control connection. Language Linguist Compass. 4(10):906–24.

[bib52] Olulade OA, Seydell-Greenwald A, Chambers CE et al. (2020) The neural basis of language development: changes in lateralization over age. Proc Natl Acad Sci USA. 117(38):23477–83. 32900940 10.1073/pnas.1905590117 PMC7519388

[bib53] Packheiser J, Schmitz J, Arning L et al. (2020) A large-scale estimate on the relationship between language and motor lateralization. Sci Rep. 10(1):13027. 32747661 10.1038/s41598-020-70057-3 PMC7398911

[bib54] Peng X, Liu Q, Hubbard CS et al. (2023) Robust dynamic brain coactivation states estimated in individuals. Sci Adv. 9(3):eabq8566. 36652524 10.1126/sciadv.abq8566 PMC9848428

[bib55] Pinheiro JC, Bates DM (1996) Unconstrained parametrizations for variance-covariance matrices. Stat Comput. 6(3):289–96.

[bib56] Pliatsikas C (2020) Understanding structural plasticity in the bilingual brain: the Dynamic Restructuring Model. Bilingualism. 23(2):459–71.

[bib57] Price CJ (2012) A review and synthesis of the first 20 years of PET and fMRI studies of heard speech, spoken language and reading. Neuroimage. 62(2):816–47. 22584224 10.1016/j.neuroimage.2012.04.062 PMC3398395

[bib58] Quackenbush J (2002) Microarray data normalization and transformation. Nat Genet. 32(4):496–501. 12454644 10.1038/ng1032

[bib59] Rousseeuw PJ (1987) Silhouettes: a graphical aid to the interpretation and validation of cluster analysis. J Comput Appl Math. 20:53–65.

[bib60] Ryali S, Supekar K, Chen T et al. (2016) Temporal dynamics and developmental maturation of salience, default and central-executive network interactions revealed by variational Bayes hidden Markov modeling. PLoS Comput Biol. 12(12):e1005138. 27959921 10.1371/journal.pcbi.1005138 PMC5154470

[bib61] Shaywitz BA, Shaywltz SE, Pugh KR et al. (1995) Sex differences in the functional organization of the brain for language. Nature. 373(6515):607–9. 7854416 10.1038/373607a0

[bib62] Stoyanov Z, Decheva L, Pashalieva I et al. (2012) Brain asymmetry, immunity, handedness. Central Eur J Med. 7:1–8.

[bib63] Vallortigara G, Chiandetti C, Sovrano VA (2011) Brain asymmetry (animal). Wiley Interdisciplinary Reviews: Cognitive Science. 2(2):146–57. 26302006 10.1002/wcs.100

[bib64] Verhelst H, Dhollander T, Gerrits R et al. (2021) Fibre-specific laterality of white matter in left and right language dominant people. Neuroimage. 230:117812. 33524578 10.1016/j.neuroimage.2021.117812

[bib65] Watanabe H, Fitting S, Hussain MZ et al. (2015) Asymmetry of the endogenous opioid system in the human anterior cingulate: a putative molecular basis for lateralization of emotions and pain. Cereb Cortex. 25(1):97–108. 23960211 10.1093/cercor/bht204 PMC4259275

[bib66] Watanabe K, Taskesen E, Van Bochoven A et al. (2017) Functional mapping and annotation of genetic associations with FUMA. Nat Commun. 8(1):1826. 29184056 10.1038/s41467-017-01261-5 PMC5705698

[bib67] Wermke K, Quast A, Hesse V (2018) From melody to words: the role of sex hormones in early language development. Horm Behav. 104:206–15. 29573996 10.1016/j.yhbeh.2018.03.008

[bib68] Westerhausen R, Kompus K, Hugdahl K (2014) Mapping hemispheric symmetries, relative asymmetries, and absolute asymmetries underlying the auditory laterality effect. Neuroimage. 84:962–70. 24121087 10.1016/j.neuroimage.2013.09.074

[bib69] Wirsich J, Giraud A-L, Sadaghiani S (2020) Concurrent EEG-and fMRI-derived functional connectomes exhibit linked dynamics. Neuroimage. 219:116998. 32480035 10.1016/j.neuroimage.2020.116998

[bib70] Wu X, Kong X, Vatansever D et al. (2022) Dynamic changes in brain lateralization correlate with human cognitive performance. PLoS Biol. 20(3):e3001560. 35298460 10.1371/journal.pbio.3001560 PMC8929635

[bib71] Xu M, Liang X, Ou J et al. (2020) Sex differences in functional brain networks for language. Cereb Cortex. 30(3):1528–37. 31512720 10.1093/cercor/bhz184

[bib72] Yang Y, Tam F, Graham SJ et al. (2020) Men and women differ in the neural basis of handwriting. Hum Brain Mapp. 41(10):2642–55. 32090433 10.1002/hbm.24968 PMC7294055

[bib73] Yeo BTT, Krienen FM, Eickhoff SB et al. (2015) Functional specialization and flexibility in human association cortex. Cereb Cortex. 25(10):3654–72. 25249407 10.1093/cercor/bhu217 PMC4598819

[bib74] Zheng B, Báez S, Su L et al. (2020) Semantic and attentional networks in bilingual processing: fMRI connectivity signatures of translation directionality. Brain Cogn. 143:105584. 32485460 10.1016/j.bandc.2020.105584 PMC7933822

[bib75] Zhou Q, Zhang L, Feng J et al. (2019) Tracking the main states of dynamic functional connectivity in resting state. Front Neurosci. 13:685. 31338016 10.3389/fnins.2019.00685 PMC6629909

